# Multiple Anomalies

**Published:** 1913-02

**Authors:** 


Multiple Anomalies.—G. Jeanneney, ibid., describes the dissection of a female cadaver, aged 74. The anomalies were: Fasiculus of trapezius inserting into sternum; division of axillary artery into radial and cubito-interosseous branches; dilatation of oesophagus; a long S-shaped hepatic tongue extending as low as the anterior superior iliac spine; azygos lobe of the right lung.

